# The Limitations of Anterior Knee Displacement during Different Barbell Squat Techniques: A Comprehensive Review

**DOI:** 10.3390/jcm12082955

**Published:** 2023-04-19

**Authors:** Gabriel Illmeier, Julian S. Rechberger

**Affiliations:** 1 Department of Strength & Conditioning, Nachwuchsleistungssportzentrum Steiermark, 8010 Graz, Austria; 2 Mayo Clinic Graduate School of Biomedical Sciences, Mayo Clinic, Rochester, MN 55905, USA; rechberger.julian@mayo.edu; 3 Department of Molecular Pharmacology and Experimental Therapeutics, Mayo Clinic, Rochester, MN 55905, USA

**Keywords:** anterior knee translation, back squat, restricted squat, unrestricted squat, knee rehabilitation

## Abstract

Based on seminal research from the 1970s and 1980s, the myth that the knees should only move as far anterior during the barbell squat until they vertically align with the tips of the feet in the sagittal plane still exists today. However, the role of both the hip joint and the lumbar spine, which are exposed to high peak torques during this deliberate restriction in range of motion, has remained largely unnoticed in the traditional literature. More recent anthropometric and biomechanical studies have found disparate results regarding anterior knee displacement during barbell squatting. For a large number of athletes, it may be favorable or even necessary to allow a certain degree of anterior knee displacement in order to achieve optimal training outcomes and minimize the biomechanical stress imparted on the lumbar spine and hip. Overall, restricting this natural movement is likely not an effective strategy for healthy trained individuals. With the exception of knee rehabilitation patients, the contemporary literature suggests it should not be practiced on a general basis.

## 1. Introduction

Since the 1980s, there has been a widespread perception that the barbell squat should be performed with an upright posture and that the knees should not be moved beyond the tips of the toes. According to this traditional dogma, anterior knee movement should be limited in the sagittal plane once a vertical line with the tips of the feet is achieved [1,2]. Widely accepted instructions for proper knee positioning during barbell squats [2,3] are based on previous studies [4,5,6] that showed that anterior knee displacement (AKD) past the toes is associated with greater shearing forces in the knees, specifically the tibiofemoral joints [4], and that moving the knees anteriorly to a lesser extent during squatting generates lower knee extensor torque [5]. Based on these findings, it has become standard practice to maintain the shin as vertical as possible and that “maximal forward movement of the knees should place them no more than slightly in front of the toes” when squatting in order to lessen the shear stress placed on the knee [3]. From a practical standpoint, these guidelines advise against allowing the knees to displace anteriorly past the toes [1,2,3]. Although not reported in the literature, the recommendation to not or only slightly push the knees over the tips of the toes seems to be a very vague statement, as disproportionately large feet in relation to the lower extremities of an individual will likely not lead to reduced shearing forces. In addition, the recommendation to limit AKD results in altered knee and hip coordination [7], with a stronger upper body inclination [2,8,9], enhanced trunk flexion in the thoracic and lumbar spine [10], and reduced squatting depth [9]. Moreover, using a moderate foot stance, which represents an outward-directed foot angle of approximately 20° with the toes pointed laterally in combination with a shoulder-width stance [11], avoiding AKD cannot be achieved by most athletes performing different barbell squat techniques [7,9,11,12]. Given that deep barbell squat variations, such as deep high-bar back squats (DHBBSs) and deep front squats (DFSs), provide several fundamental benefits, including greater muscle activation, improved functional capacity, and higher athletic performance, as well as performance-enhancing transfer effects of dynamic maximal strength to dynamic speed-strength of hip and knee extensors [13,14,15], they are likely to be preferred over variations where the range of motion is deliberately limited under most circumstances. The center of gravity must remain vertically above the supporting surface during all barbell squat techniques, otherwise balance cannot be maintained [10,16]. To ensure this, anthropometry and biomechanics require the majority of exercisers to move the knee joints anteriorly over the toes during deep barbell squats. This is considered a normal and a required part of the squat movement, which should be encouraged in healthy individuals [17]. Even more so, the American College of Sports Medicine (ACSM) advised that healthy adults should perform every exercise through a full range of motion [18]. Exercising over the entire range of motion enables strength adaptations to take place at every angle the joint traverses, which may lower the risk of injury in those ranges [19]. Conversely, it has been demonstrated that spinal flexion and extension have a major impact on joint kinetics when performing squats [20]. To lower the risk of lumbar spine injuries, it is crucial to maintain a neutral spine position when lifting objects [21]. Practice guidelines concur that, in order to lower the risk of injury, both the spine and pelvis should remain in a neutral position with no relative movement during the squatting motion [20,22,23]. This is particularly true at the bottom position of the squatting movement, when the maximal angular displacement of the trunk and pelvis is greatest [21]. Deep squatting therefore seems to be a safe exercise when a neutral spine is maintained, although AKD increases with the depth of the squat [7].

In this comprehensive review, we summarize the contemporary literature and discuss the role of AKD during different variations of barbell squatting for either exercise, rehabilitation, or therapeutic purposes. It should be noted that AKD is only quantified or addressed in a few studies and is rather a by-product of the actual research question in most studies. Moreover, to the knowledge of the authors, there was no study or review conducted that addresses AKD in its complexity and full depth. The goal of this review is to clearly present all relevant factors that influence AKD when barbell squatting and to establish cross-connections that may not be apparent at first glance. For this purpose, relevant findings from anatomy, anthropometry, and biomechanics are compiled. Moreover, there are few studies that deal with anthropometry in the context of barbell squatting. Furthermore, to the best knowledge of the authors, there is no study that examines the role of AKD during different barbell squat variations or, rather, how much AKD is needed for optimal squatting when performing different techniques.

Traditional technique guidelines for barbell squats and generalized recommendations regarding AKD are a subject of increasing controversy in the pertinent area of research, and correspondingly, the fact that AKD is by no means harmful but actually beneficial for the barbell squat movement pattern in healthy athletes is meticulously reviewed. This is significant because it is critical to inform the readership of the research landscape to date and future directions of this active area of investigation to provide the best possible support for athletes and patients alike.

## 2. Materials and Methods

The search strategy was designed to capture all possible indexed articles in online databases referring to anterior knee movement during different barbell squat variations. PubMed, ResearchGate, and Google Scholar were searched for referenced articles from their date of inception until April 2022. All databases were searched and screened independently by two investigators (G.I. and J.S.R.) using the following string of search terms: “restrict* squat* AND unrestrict* squat*”, “restrict* squat* OR unrestrict* squat*”, “anterior tibial translation AND squat*”, “anterior knee displacement AND squat*”, “anthropom* AND squat*”, “anterior knee travel AND squat”, “restrict* ankle dorsiflexion AND squat*”, “leg length AND squat*, “segment length AND squat*”, “squat* AND biomechanic*”, “squat* AND spine”, “barbell squat*”, and “squat*” and checked for relevant articles for inclusion in the review. In addition, a direct search of the Journal of Strength and Conditioning was conducted to identify additional publications. Publications that addressed AKD in the context of anthropometric or biomechanical studies of different barbell squat movements for exercise, rehabilitation, or therapy were included in this qualitative literature review. Publications were limited to the German and English languages. The findings from the selected publications were summarized and relevant relationships between the AKD, barbell squat biomechanics, and anthropometry were identified. Based on the results, practice-relevant recommendations were formulated.

## 3. Results

### 3.1. Restricted vs. Unrestricted Barbell Squats

From a biomechanical perspective, AKD past the toes during squatting is associated with greater shearing forces on the knee [4]. It was therefore suggested that the resulting knee excursion could contribute to knee injury and that the shin should be kept as vertical as possible in order to reduce the shear stress on the knee [3,4]. However, the impact on the hip joint and the lumbar spine was not considered by these authors.

Research by Fry et al. [2] confirmed that knee torque increased by about 28% on average when exercisers performed unrestricted parallel high-bar back squats (PHBBSs; knee torque unrestricted squat 150.1 ± 50.8 Nm vs. knee torque restricted squat 117.3 ± 34.2 Nm), which are defined by a knee angle of 60–70° [16] and the knees were allowed to move beyond the toes. It was previously reported that patellofemoral joint stress (PFJS) during squatting increases during the lowering phase, continues to slightly increase during the rising phase, and finally decreases as the knee becomes more extended [24]. Additionally, peak PFJS and patellofemoral joint reaction forces rise as AKD, knee flexion angle, and external resistance increase [24,25]. The force of the quadriceps muscle might lead to anterior tibial displacement, particularly close to complete knee extension [26]. This displacement is counteracted by the quadriceps/hamstring co-contraction during squatting [27,28,29,30,31]. It is assumed that this co-contraction helps to neutralize the tibiofemoral shear forces imparted by the quadriceps [31], thus providing a stabilizing force at the knee during squatting [28].

Conversely, it was also shown that the external torque on the hip joints increased by approximately 973% (hip torque unrestricted squat 28.2 ± 65.0 Nm vs. hip torque restricted 302.7 ± 71.2 Nm) when the AKD was deliberately limited to toe-off height [2]. Biomechanically, this is an enormously high torque on the hip joint and likely much less favorable than the slightly increased torque on the knee joints. Assuming a simple kinematic chain model with a similar ground reaction force at the deepest position, the approximately 10-fold larger observed momentum in the hip during restricted high-bar back squats further suggest higher torque on the lower back [2,9]. The distance related to the moment of the muscle force around the screw axis of the joint is known as the moment arm, which has been demonstrated to increase in the hip joints when restricting AKD [2]. Conversely, a greater dorsiflexion of the ankle joints, which results in larger moment arms for the ground reaction force, explains why the maximum moment in the knees during unrestricted squats is greater than that during limited squats with larger knee flexion angles [9]. The idea of the moment arm appears to be crucial in practice, as the LBBS is particularly recommended when the primary goal is to lift weights as heavy as possible [32]. By placing the barbell lower on the back, the LBBS decreases the moment arm in key anatomical compartments and facilitates enhanced biomechanical working conditions for the hip extensor muscles, ultimately allowing for heavier weights to be used during squatting [33]. While in LBBSs, the moment arms in the hips are greater than when performing high-bar squats; the latter exercise affords moment arms in the knees that are relatively higher than in the hips [34].

What is more, the difference in maximum hip momentum during restricted and unrestricted high-bar back squats increases with increased barbell load, being 6.9% higher for restricted high-bar back squats with bodyweight only, 11.3% higher with one and one-quarter of the bodyweight, and 14.6% higher with one and one-half of the bodyweight [9]. It was therefore concluded that although deliberate restriction of the AKD lowers the torque on the knee, disproportionately high forces occur in the hips [2], which are likely transferred towards the lower back [2,9]. Adequate joint loading during barbell squatting consequently requires movement of the knees beyond the toes [2,16]. 

Restricting the natural AKD leads to altered knee–hip coordination [6], which is associated with increased trunk flexion in the thoracic and lumbar spine [10]. This form of evasive movement can lead to increased tensile stress on the intervertebral ligaments [35,36] and has been consequently discouraged by various authors [37,38]. It was demonstrated that the torque in the knee joints is significantly higher in the unrestricted high-bar back squat (maximal knee flexion angle of 85 ± 11°) than in the restricted high-bar back squat (maximal knee flexion angle of 106 ± 10°) [9]. However, the resulting torque in the hip joints behaved in an inverse manner. The higher torque in the hips with restricted high-bar back squats indicated a higher load on the lower back, which is why the authors argued that unrestricted high-bar back squats should be the preferred technique. Accordingly, subsequent studies found that unrestricted barbell squat variations are more suitable for stimulating the lower extremities [39], minimizing strain on the lower lumbar spine and lower back in comparison with restricted techniques [8,9], and are therefore the recommended technique to improve athletic performance based on current evidence [40].

### 3.2. Barbell Squat Technique Variations and AKD

**Figure 1:**
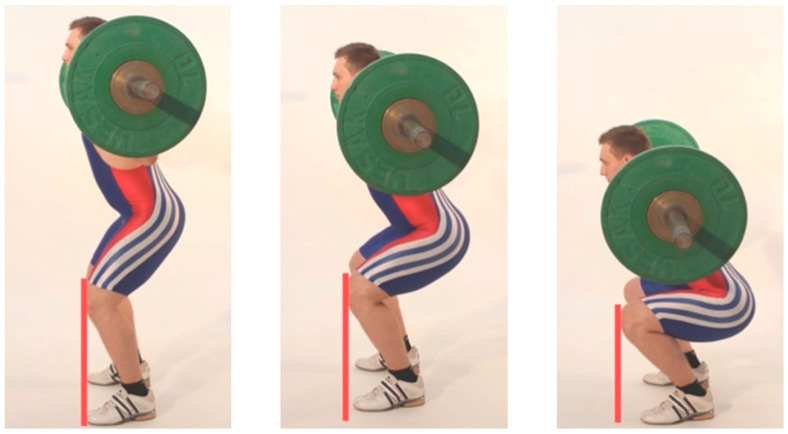
“Quarter”, “half”, and parallel high-bar back squat (QHBBS, HHBBS, and PHBBS). AKD is enhanced by lower squatting depth (see red lines). Modified with permission from [38].

To avoid AKD, without performing a technically inaccurate barbell squat in which the peak loads shift towards the lumbar spine, reducing the depth of the squat is an applicable strategy. While in clinical settings full knee extension is typically defined as 0°, the studies discussed here utilize a 180° knee angle to define full knee extension (i.e., 180° being equivalent to a straight stance) [41]. Accordingly, in “quarter” high-bar back squats (QHBBSs) and “half” high-bar back squats (HHBBSs, defined by a knee angle of approximately 110–140° and 80–100°, respectively), the knees of most athletes are not or only slightly pushed anterior over the toes (see Figure 1, left and center) [13,41,42].

Subjects performing a QHBBS were able to move an average load 4.02 (±1.59) times greater than with a DHBBS (defined by a knee angle of approximately 40–45°) [13,14,34,38]. However, such weights are not used in routine training practices for advanced athletes given that such high loads cannot be stabilized by the trunk or spine. Furthermore, with heavier weights, the compression forces on the vertebral bodies and the intradiscal pressure on the intervertebral discs also increase [43,44]. Heavier weight loads also lead to a significant augmentation of tibiofemoral [26] and patellofemoral compression forces [25]. As reviewed by Hartmann and Wirth [38], these relationships are often not considered when examining spinal [20] and knee joint loads at different knee flexion depths [20,45]. QHBBSs and HHBBSs with relatively supramaximal loads as compared with DHBBSs increase the risk for long-term degenerative alterations in knee joints and the spine [41]. Contrary to frequently expressed concerns, it has been shown that DHBBSs present an effective exercise for protection against back and hip injuries and do not contribute to an increased risk of knee injury [41], even though the AKD has been reported to range from 63.8 to 64.7 mm in men when squatting to a knee angle of 59.1 ± 2.0° and 93.2 to 96.6 mm in women when doing so to a knee angle of 72.4 ± 2.9° [7]. Taken together, these studies indicate that eliminating or minimizing AKD by reducing the squat depth with QHBBSs and HHBBSs may not be the most optimal choice for healthy individuals aiming to strengthen the muscles of the lower extremity. It should be noted, however, that squatting with a range of motion between 180° (upright stance) and 130° of knee flexion has been shown to minimize patellofemoral compressive forces [46] and may therefore be an appropriate option for knee rehabilitation patients, using no or very little additional load. A flexion depth of 130° corresponds to a “quarter” squat (QS) where the knees do not move past the toes (Figure 1, left). In this functional range, patellofemoral compressive forces and tibiofemoral compressive and shear forces in the knee joints are minimal [45], and deliberate limitation of AKD can therefore be an adequate strategy for rehabilitation [24].

### 3.3. Barbell Placement and AKD

**Figure 2:**
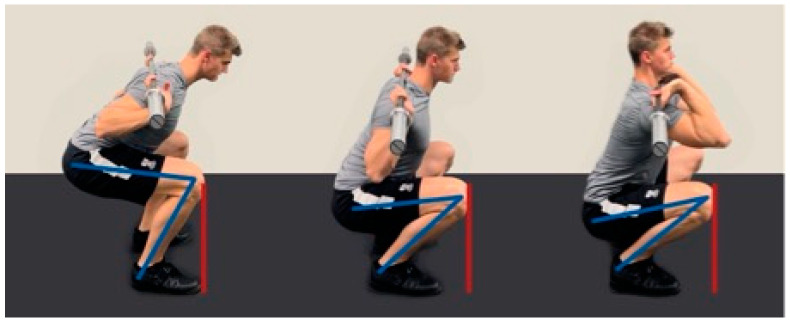
Squat variations and AKD: Low-bar back squat (LBBS, **left**), deep high-bar back squat (DHBBS, **center**), and deep front squat (DFS, **right**). AKD (red lines) and knee angle (formed by the lines in blue) vary between different barbell squat techniques.

AKD is directly related to the positioning of the barbell on the neck, as differences in the placement of the bar lead to a change in the overall center of gravity of the body [33]. Consequently, in order to maintain balance in an upright position, the positioning and joint angles associated with the lower extremities and the trunk also change. The more anteriorly the barbell is placed, the more upright the upper body can be positioned during a squatting movement. A more upright upper body or a greater trunk segment angle (TSA) afforded by a DFS (TSA: 63.6 ± 4,2; Figure 2, right) and a DHBBS (TSA: 46.3 ± 4,8; Figure 2, center) is associated with greater AKD than a low-bar back squat (LBBS—Figure 2, left), which has a more restricted relative to horizontal TSA of 40.7 ± 5.8 [16]. Moreover, a DHBBS allows for a greater degree of squatting depth than a LBBS because it permits more knee flexion, and this also impacts AKD (Figure 2) [38,47].

### 3.4. Ankle Mobility, Weightlifting Shoes, and AKD

A sufficient degree of ankle dorsiflexion (ADF) forms the basis for a technically correct squat, regardless of the specific variations in technique [48]. Limited ankle mobility has been demonstrated to have a negative effect on squat exercise biomechanics [49,50] and, specific to young athletes, might be a significant contributor to the development of Osgood–Schlatter disease [51]. The ankle joints therefore need to have adequate closed kinematic chain range of motion to meet the technical requirements for lowering and elevating the center of mass vertically [37,52]. When performing a barbell squat, a sufficient amount of ADF is most important at the bottom of the descent phase [53]. As the hips, knees, and ankles flex, limitations in ankle flexibility and the consequently limited amount of AKD may arise as compensatory movements as complete joint range of motion is reached [7]. Numerous studies have revealed that the greatest restriction for compensating squatting mechanics is the ankle joints [48,54,55,56]. While attempting to lower the center of mass during the descent phase of the squatting movement, an individual may develop compensatory movements due to constraints in their ADF range of motion (ADF-ROM) [56]. It has been demonstrated that greater active ADF-ROM is associated with greater knee flexion and ADF displacement during squatting and that simulating reduced ADF with a wedge (12° forefoot angle) decreases peak knee flexion [48,54]. Moreover, it was shown that average ADF was 23.4–25.9° (measured as the angle between the line connecting the fibula head and the calcaneus and the line connecting the base and head of the fifth metatarsal bone) when deep bodyweight squats (56°) were performed and suggested that ADF is an important factor that determines the ROM of deep squats (DS) [57].

Accordingly, it was shown that ADF is crucially implicated in determining squatting depth [57] and that deep squatting requires a large range of motion of ADF [58]. Since the athlete’s center of mass is primarily lowered by knee flexion [56], other joints in the kinetic chain must make up for this in order to complete the movement [59]. As a result, increased peak knee valgus angle [53,60,61] and altered spinal alignment [37] have been reported during squatting when there is reduced ADF-ROM.

The degree of maximum ADF has been shown to differ between men and females and is associated with the depth of the barbell squat [7,62]. The resulting combination of squat depth and possible ADF significantly impacts AKD (see Figure 1), which has been reported to peak at 84% and 93% of the maximum depth during squatting in men and females, respectively [7]. Partial range of motion squatting movements, such as QHBBSs, demand less joint displacement throughout the lower extremity relative to deeper squatting techniques, such as PHBBSs [63]. The more anteriorly a load is placed, the more ankle mobility and AKD were required for correct exercise execution (Figure 2). A DFS (Figure 2, right) therefore requires more ADF than different back squat variants (Figure 2, left and center) and was consequently associated with more AKD than a DHBBS or a LBBS (Figure 2, lines in red).

In their 2012 study, Sato et al. compared the kinematics of PHBBSs performed while wearing running shoes and weightlifting shoes [64]. When the latter shoes are used, the elevation of the heel compared with the forefoot is most commonly set around 2.5 cm and simulated a greater amount of knee dorsiflexion range of motion. The authors discovered that wearing weightlifting shoes while squatting resulted in a greater plantar flexion angle and less forward leaning of the trunk. Although the mean differences between groups were small (3.5°), there was a large effect size (0.72), suggesting weightlifting shoes likely facilitate more plantarflexion when the leg segment is vertically aligned at the start of the squat. Previous research using ankle position that starts in plantarflexion to aid the squatting movement (similar to the effect created by the weightlifting shoes) indicates an increase in lower extremity muscle activation, particularly in the knee extensor muscles, which is similar to the effect produced by wearing weightlifting shoes [65]. Since weightlifting shoes are expected to have a greater drop, they may consequently lead to a greater involvement of the knee extensor muscles during the squatting motion when compared with running shoes. What is more, a larger foot segment angle, which is defined by a hypothetical plane passing horizontally through the ankle joints in the frontal plane and parallel to the ground, produced by wearing weightlifting shoes may, to a certain extent, permit a more pronounced AKD and may be advantageous for individuals looking to strengthen their knee extensor muscles [64]. In concordance with previous results, it has also been shown that weightlifting shoes permit a greater degree of upright posture with more knee flexion during squatting [66]. Furthermore, they decrease trunk lean and generate more plantar flexion relative to running shoes and barefoot lifting [67]. Elevating the heel through the use of external squat wedges is a popular method during rehabilitation and was shown to provide similar effects to weightlifting shoes [66,67].

### 3.5. Anthropometrics, Foot Placement, and AKD

**Figure 3:**
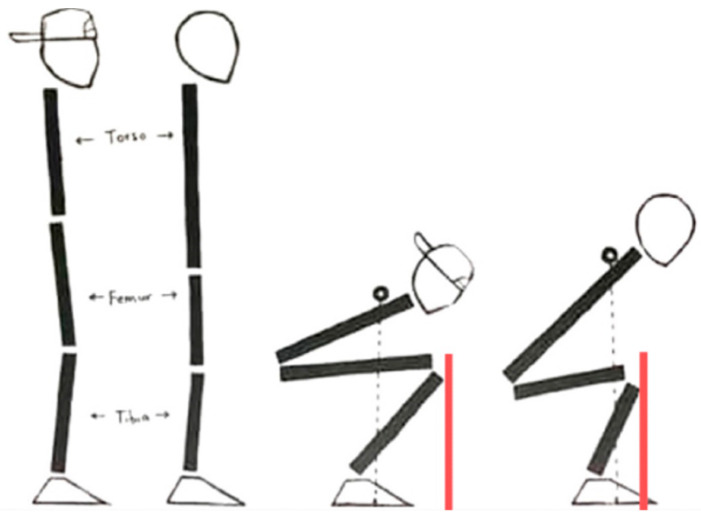
Anthropometry and AKD during squatting—(**left**): long legs/short torso vs. (**right**): short legs/long torso. Limb-to-torso-ratio affects AKD when squatting (red lines). Modified with permission from [70].

Anthropometrics, in particular the various length ratios of the body, as well as stance width affect upper body positioning and AKD during squatting [68]. Athletes with relatively short legs and a long torso are able to minimize AKD when employing a proper technique (Figure 3, right). In contrast, athletes with relatively long lower extremities and a short torso are required to move their knees further anterior (beyond the tips of the toes) to stay balanced during the entire movement (Figure 3, left) [37]. A wide stance width, due to the effective shortening of the thigh in the sagittal plane and the resulting favorable thigh-to-lower-leg-ratio, decreases the required range of ADF which, when reduced, limits AKD during squatting [68]. Moreover, squatting patterns diverge between genders, which may be a result of differences in limb length and limb-to-torso-ratio between men and women [69]. Underlying anthropometric differences consequently impact AKD (Figure 3) [37].

Moreover, it has been shown that in the “sticking region” of a DHHBS (54.7 ± 6.6°), which starts in the ascending phase at 64.8 ± 6.9° knee flexion, the hip moment arm and hip contribution to total moment increase, whereas the knee moment arm and moment contribution to total moment decreases, indicating a load shift towards the hips [71]. With regard to this, it was speculated that the sticking could be knee flexion angle specific because less force can be produced due to large external moments and moment arms in combination with unfavorable biomechanical working conditions for the gluteus maximus [71,72]. Furthermore, at more than 50° knee flexion, a 6° difference in knee flexion does not significantly change the length of the moment arm to the center of mass of the body, although a reduction of the sagittal moment of 43–63% with a foam knee support was found [73]. Similarly, a 59% reduction in calculated compressive knee force due to thigh–calf contact was found for a deep bodyweight squat (25°) [74], which is an often overlooked and a not well studied topic when it comes to knee joint contact force prediction [75].

## 4. Discussion and Limitations

Due to the widespread use of the barbell squat in fitness, rehabilitation, and therapy, various techniques and modifications have been established by altering the squat bar position: high-bar back squat (barbell placed across the back slightly above the acromion), low-bar back squat (barbell placed across the back slightly below the acromion), front squat (barbell held in front of the chest approximately on the clavicles and above anterior deltoid), and overhead squat (barbell in an overhead position while elbows are fully extended and radial-ulnar joints pronated) [76,77,78,79]; modifying the squat depth: partial squat (representing a squatting depth less than parallel), which semantically includes quarter squats (QSs) (110–140° knee angle) and half squats (HSs, 80° to 100°), parallel squats (PSs) where the thighs are parallel to the floor (60–70°), and DSs or full squats (FSs), distinguished by a squat with thighs deeper than parallel, where knee angles have been shown to reach as much as 40–45° knee flexion [7,19,34,38,80,81,82]; modifying the stance width and foot rotation: narrow stance (107 ± 10% of the shoulder width), medium stance (142 ± 12% of the shoulder width), or wide stance (169 ± 12% of the shoulder width) with internally or externally rotated foot position [11,83]; or modifying the squatting surface: stable or instable underground (e.g., power board, BOSU ball, and balance cone) [79].

However, there are currently no widely accepted standard methods for quantification of the abovementioned techniques and different studies may use different terms [20]. Given the current research landscape, a consistent definition of semantics pertaining to all common barbell squat techniques, including a unified angle convention, would help to avoid confusion. AKD must always be seen in the context of the particular squat technique used, which is impacted by various parameters. Partial squats, QSs, HSs, PSs, FSs, and DSs are names that are present in the literature and lack a consistent, standardized definition. As a result, in previous studies, the same squatting techniques were frequently referred to using different expressions by different authors and vice versa when different techniques were referred to using the same names. For example, Schoenfeld [20] defined the start and end position of the squat, where the knees are fully extended, at 0° in comparison to 180° as used by McKean et al. [7], Hartmann & Wirth [38], and many other authors, including those of the current review. Consequently, the latter terminology was used for this review. Schoenfeld categorizes the squat into three basic groups by measuring the posterior angle between the thigh and the lower shank: partial squats (40° knee angle, which corresponds to a 140° knee angle according to the angle convention system used in this review, where the start and end position of the squat, with fully extended knees, is considered 180° and not 0°), HSs (70° to 100° knee angle, which corresponds to 80–110°), and DSs (greater than 100°, which corresponds to a squat deeper than 80°) [20]. In addition to the potential confusion that may occur due to the difference in definitions of the starting angle, which at first glance could tempt confusion of a partial squat with a DS or FS, the designations of the knee angle range “partial”, “half”, and “full”, even after conversion, do not correspond to those of other authors; for instance Fry’s et al. [16] PS (60–70°) already being a FS (deeper than 80°) according to the stated definition of knee angles by Schoenfeld [20].

In order to solve this problem, the authors of the present review suggest the following approach:

1. Defining the barbell squat variations by the placement of the barbell (front squat, high-bar back squat, low-bar back-squat, overhead squat) and avoiding imprecise expressions such as back squat.
2. Defining each squatting technique by a common name that corresponds to its depth or knee angle range as suggested by various authors (partial squat: QS/HS, PS, FS/DS) plus adding the exact knee angle range examined and unifying the measurement method (e.g., defining the start and end position of the squat, where the knees are fully extended, as 180° and measuring the posterior angle between the thigh and the lower shank and not the supplementary angle).
3. Adding photos (lateral and frontal view) where the start and end positions are shown and the placement position of the barbell is visible.
4. Adding a figure where the angle conventions used for analysis of the study are clearly explained and visible, as prepared by McKean et al. [7].

By doing so, the definition of a DHBBS (40–45°) or parallel LBBS (60–70°), further including information on whether a barbell is used, where it is placed, and how deep the squats were performed, includes vital information that allows the reader to precisely understand what squatting technique is employed. Based on the provided information, AKD and its role for each individual athlete or patient can be better investigated.

In summary, traditional technique guidelines for barbell squats and generalized recommendations regarding AKD should be critically questioned. Individual anatomy, anthropometry, and barbell placement all contribute to the resulting movement patterns during different barbell squat techniques. Although the deliberate restriction of AKD can reduce the torque on the knees, traditional recommendations of AKD result in altered knee and hip coordination [7], with a stronger upper body inclination [2,8,9], enhanced trunk flexion in the thoracic and lumbar spine [10], and consequently, disproportionately high forces transferred towards the hip joints and lower back. Additionally, most athletes employing various barbell squat techniques cannot prevent AKD when using a moderate foot stance, which is indicated by an outward-directed foot angle of roughly 20°, with the toes pointed laterally in conjunction with a shoulder-width stance [7,9,11,12]. As deep barbell squat variations, such as DHBBSs and DFSs, offer a number of fundamental advantages, such as increased muscular activation, improved functional capacity, superior athletic performance, and performance-enhancing transfer effects of dynamic maximum strength to dynamic speed-strength of hip and knee extensors, these techniques are likely to be chosen over variations where the range of motion is deliberately limited [13,14,15].

In order to maintain balance, the center of gravity during all barbell squat techniques must remain vertically above the supporting surface [10,16]. Anthropometry and biomechanics dictate that most exercisers must perform deep barbell squats with their knees anteriorly over their toes in order to achieve this. This is seen as a typical and necessary component of the squatting movement, which healthy individuals should be encouraged to perform [17]. A restriction of movement depth to QSs and HSs in order to avoid or minimize AKD has been demonstrated to be suboptimal for healthy individuals strengthening the muscles of the lower extremity, as subjects performing a QHBBS were able to move an average load 4.02 (±1.59) times higher than with a DHBBS [14]. Such loads, however, cannot be used in routine training practices for advanced athletes given that they cannot be stabilized by the trunk or spine. Conversely, the deliberate limitation of AKD may present an adequate strategy for knee rehabilitation patients, as it has been shown to minimize patellofemoral compressive forces and shear forces in the knee joints. For healthy individuals, however, a pronounced AKD during squatting poses no health risks and should not be deliberately limited based on current evidence.

## 5. Conclusions

Studies on the anthropometric and biomechanical aspects of AKD during barbell squats have produced conflicting findings. In order to attain the best training results and reduce the biomechanical stress placed on the lumbar spine and hip, it may be advantageous or even required for many athletes to permit a certain amount of AKD. Overall, limiting this natural mobility is probably not a good idea for healthy, trained individuals. The most recent research indicates it should not be used routinely, with the exception of knee rehabilitation patients.

## Data Availability

Not applicable.
